# Promoting healthy eating in primary health care from the perspective of health professionals: a qualitative comparative study in the context of South America

**DOI:** 10.1186/s40795-018-0244-9

**Published:** 2018-10-25

**Authors:** Alexandra Pava-Cárdenas, Kellem Regina Rosendo Vincha, Viviane Laudelino Vieira, Ana Maria Cervato-Mancuso

**Affiliations:** 1 Nutrition Department, School of Public Health, University of São Paulo, Av. Dr. Arnaldo, 715 –, São Paulo, SP 01246-904 Brazil; 2 Paula Souza Health Center, School of Public Health, University of São Paulo, São Paulo, Brazil

**Keywords:** Nutrition, Health systems and services in low and middle-income settings, Primary care, Health promotion/prevention/screening, Qualitative research

## Abstract

**Background:**

Educational interventions designed to promote healthy eating are essential in primary health care. Nevertheless, given the nutrition controversies about what is healthy, the contradictions created by the media, and the situation of users with complex needs, the prioritization of the themes to be addressed in the services has scarcely been described in the planning process. This study aimed to identify the process of implementing the themes discussed by health professionals in nutrition education groups in two primary health care models.

**Methods:**

Our study followed a qualitative comparative approach. It included the systematic observation of nutrition education group meetings to identify the key messages addressed and semi-structured interviews with health professionals in São Paulo, Brazil, and in Bogotá, Colombia. We used thematic networks to classify the messages and the collective subject discourse technique to organize the information obtained from interviews. We observed 28 nutrition education groups in São Paulo, and 13 in Bogotá, and conducted 27 interviews with nutritionists in each city.

**Results:**

The messages identified were grouped into four global themes: feeding habits, life cycle, disease, and “being a multiplier”. The process of implementing the themes, understood as identification, selection, consultation, and application of themes, is intermediated by social representations of the health professionals about service requirements, training and professional performance, and the relationship with users. Two notions shape these representations: Control, although the time and the physical space dedicated to health services are restricted to the disease in São Paulo, in Bogotá only limited health promotion is provided; and specificity, which is portrayed as therapeutic support within a more educational model in São Paulo and as health promotion training courses within a prescriptive model in Bogotá.

**Conclusions:**

Understanding the process of implementing the themes discussed in nutrition education groups can reveal mechanisms that support the approach to themes on healthy eating, including communicative and educational adaptations of health professionals. This study contributes to the discussion about educational models in health care and their effects on the qualifications of health professionals within the service*,* especially those included in the context of low- and middle-income settings*.*

## Background

The nutritional transition in low- and middle-income countries (LAMICs) corresponds to a phenomenon different from that experienced in developed countries [1]. The divergences are related to the speed of economic, political, technological and social transformations [2]. However, the coexistence of under- and over-nutrition in different social groups and the variation of policy environments to cope with the rapid increase in the prevalence of noncommunicable diseases are particularities [2, 3]. On the one hand, developed countries have invested in the reduction of exposure to tobacco and alcohol consumption, improvements in diet, programs to promote physical activity and better treatment of chronic diseases. On the other hand, in LAMICs, the implementation of nutrition policies focuses on fighting undernutrition, although nutritional interventions for overweight persons are challenging in the current reality [3].

Evidence has indicated the importance of undertaking educational action in Primary Health Care (PHC), which has greater relevance in LAMICs [4, 5]. Specifically, good evidence has been associated with the prevention of type 2 diabetes, particularly directed to eating habits, physical activity, and overweight and obesity [6]. Therefore, in order to deal with nutritional transition challenges, Nutrition Education (NE) requires health promotion and disease prevention to be developed through participatory and critical education [7, 8].

Given the political incentive for NE, it is important to understand the effectiveness of interventions in PHC, which can be specified and measured by characteristics of structure (policy level), process (health services), and health outcomes [9]. However, professionals and researchers emphasize the results [10] rather than the process, that is, planning and the way it occurs within a health structure. Moreover, when described, the results are generalized, making it impossible to understand the scope of the interventions [11].

Experts in nutrition and behavioral sciences, including scientists and communicators, have the potential to conceive and communicate scientific knowledge about nutrition to promote healthy eating and prevent chronic noncommunicable diseases [12]. There is a tendency towards nutritional messages focusing on the reduction of fat intake and increase of fruit and vegetable intake, but little is known about the phenomenon of communication and the efficacy of these messages, especially in public health and nutrition [13]. It is understood that for messages to lead to changes in the eating habits of individuals, NE interventions must be planned and performed by professionals based on evidence or professional practice [13, 14].

Conceptions about food practices and their relationship to health have been developed, with an emphasis on measurement, prediction, and systematic organization of diets to enhance health and avoid disease [15]. Additionally, qualitative studies reveal a wide range of meanings associated with health interventions, where people regard healthy food and ways of eating to be monotonous, not tasty, and not satisfying [16]. In this way, the challenge in NE interventions is to involve the multiple perceptions about diet and health conditions of users, which are competing sources of information available in different media [17] . This has an impact on the social meanings of what health is and on care for the development of educational messages [18, 19]*.*

In the Latin American Region, comprised of LAMICs and considered as the most unequal in the world [20], there is also a lack of theoretical bases for NE [21]. This is reflected in the scarce pedagogical descriptions of interventions, leading to the lack of a specific theory of work the promotion of healthy eating [21, 22]. Within this perspective, NE includes a transforming communicative process, which involves the dimensions of learning of all individuals or groups that become the subjects in an exchange relationship [8, 23]. According to Paulo Freire, a role model of popular education, education is essentially understood as a liberating practice, which involves a joint search for specific content performed by both educator and students [24]. Health professionals, through their link with the population, facilitate the construction of a dialogue and the process of reflection and action in such a way as to encourage autonomous participation to promote critical thinking of users, a favorable approach to achieving healthy eating [8, 23, 24].

To ensure the effective of NE intervention, health promotion seeks to include the improvement of the health care workforce’s abilities related to professionalism, encompassing both theoretical and practical understanding [25, 26]. Grasping the existing need and priorities of themes or public health messages is the starting point for the educational intention [24], because a key practice task contributes to the development of appropriate and contextualized NE interventions. It is impossible to understand these themes without including the subjects involved.

Qualitative comparative studies in the international sphere are becoming more important in research, because they make broader social focuses and contrasts possible. This makes more sense for the understanding of the different experiences found in two contexts, where it is not possible to control the variables, but rather to follow certain elements of comparison [27]. Thus, to understand the selection of the themes of NE interventions included in health services and interpreted and translated by health professionals, we sought to understand the process of implementing the themes discussed by health professionals in NE groups in two PHC models.

## Methods

We performed a qualitative study, whose methodological procedure consisted of a comparison of two contexts, those of the cities of São Paulo and Bogotá, the respective economic centers of Brazil and Colombia. Both Brazil and Colombia have been experiencing similar and rapid demographic, epidemiological, and nutritional transitions, with a growing prevalence of overweight [28, 29]. To contextualize the panorama of the countries for the decade 2000–2010, the malnutrition estimates of children aged 0–59 months correspond to: Wasting–moderate and severe: 1.6 and 0.9%; Stunting 7.1 and 12.7% and Overweight: 7.3 and 4.8% [5].

However, the countries have different PHC policy interpretations and distinct stages of implementation [30]. In Brazil, the emphasis is placed on social medicine and research in collective health, integrating medical care with health promotion and public health actions. Its health system has recently strengthened PHC, which has become a new link between multidisciplinary team members and the population. After the Family Health Program (Portuguese acronym: PSF) was created in 1994, the Family Health Support Center (Portuguese acronym: NASF) was established in 2008, which has spread nationally with coverage integrated into Health Centers [31].

Colombia, although already having isolated PHC experiences in its various regions, has recently experienced a national consolidation of policies to strengthen the health system through PHC and emphasizes health promotion in the 10-year public health plan [30]. Bogotá, the capital city, has been a reference point: its renovation started in 2004 and the implementation of the Collective Intervention Plan (Spanish acronym: PIC) acts through a structure that combines management, surveillance, and operation of actions in real life scenarios within the public health field [32]. These, in turn, relate to the environments where people live, grow, and interact: family, school, community, workplace, and the institutions that provide health care. Every scenario has a different kind of program and is managed by an appropriate multidisciplinary team.

### Sample and recruitment

The research participants in this study were health professionals, specifically nutritionists, from the NASF in the city of São Paulo and from the PIC in the city of Bogotá. We approached the Department of Health (DH) to obtain a list of health professionals. Nevertheless, given the administrative diversity in health between the regions, it was necessary to map the professionals with the help of key informants by city: in São Paulo, through public social health organizations, and, in Bogotá, through the DH. The key informants authorized the participation of health professionals and signaled the professionals to be invited.

São Paulo was divided into five sub-regions, administered by public social health organizations [33], and Bogotá, into 20 areas managed by 14 public hospitals responsible for public health. We implemented this procedure to encompass the various social contexts of this city, because contrasts were sought. Subsequently, we tracked health professionals and selected those who were willing to participate voluntarily, with their free and informed consent, according to convenience so as to obtain the participation of at least one from each administrative region. Our specific inclusion criteria were: 1) to lead NE groups, 2) to work in PHC, 3) to have at least 1 month of experience on the job, 4) to voluntarily agree to participate in the study and 5) during group observation, users agreed to authorize the use of written records of the meetings.

Participants included 54 health professionals, 27 from each city. They filled out the general identification form on the Internet and were interviewed at their workplaces (Table 1). In all, 41 group meetings were observed: 28 in São Paulo, average group size of 12, including 341 users, and 13 in Bogotá, average group size of 7, including 183 users.

**Table 1:** Characteristics of health professionals and their groups in Primary Health Care. São Paulo, Brazil, and Bogotá, Colombia, 2012

Characteristics	São Paulo (*n* = 27)	Bogotá (*n* = 27)
General identification of professionals	Between 24 and 45 years of age, exclusively female.	Between 22 and 55 years of age, included 4 males.
Training of professionals	All had postgraduate qualifications.	Six reported having postgraduate qualifications.
Length of experience of professionals	Between 6 months and 4 years of experience on the job.	Between 1 month and 15 years of experience on the job.
Work routine of professionals	Individual consultations, therapeutic groups, home visits, team meetings and interviews, and support and advice for health teams.	Held workshops for people, made home visits, lead meetings and wrote reports on the intersectional Food and Nutrition Surveillance System.
Conformation of the nutrition education groups	Groups were organized based on health according to the service, the epidemiological information and demands from health centers. Focused on diseases such as obesity, diabetes, and hypertension or on the life cycle of specific groups, such as pregnant women, infants, children, parents and children, women and adolescents.	Groups were formed by popular initiative and health professionals worked with different institutions in a certain area. The number of meetings established and focused on the “You Are Valuable,” “Move On,” and “Maternal and Child Network” (*Tú Vales, Muévete, Red Materno Infantil)* programs, generally consisting of pregnant and lactating women, the elderly, multipliers, and teachers.
Development of the nutrition groups	Group meetings lasted between 1 and 2 h, were held weekly, biweekly, or monthly, were attended by 2 to 15 users, were both open and closed, and had a previously undefined number of meetings.	The meetings lasted between 1 and 3 ½ hours, were held weekly, biweekly, or monthly and the groups were attended by 3 to 31 users. The groups were closed, and the number of meetings was pre-established.

### Data collection

We established the credibility of the data by including researchers previously trained in São Paulo to conduct interviews and act as observers for 1 month. Additionally, we developed a procedural protocol. Between July and December 2012, three trained researchers collected data in the two cities: two researchers were in São Paulo and one was in Bogotá. The researcher responsible for Bogotá was fluent in both Spanish and Portuguese.

The search for contents reported in this study is understood as the process of implementation of themes, which includes the identification, selection, consultation and application of such themes. We used three approaches: a systematic observation, a semi-structured interview and an online questionnaire. Initially, the first two authors, individually, conducted the systematic observation. They used as the methodological basis, focusing on the identification of the topics, clarity and language, and key messages that were addressed in the NE groups and registering the details of the different contexts. The researcher attended the meeting of one NE group to observe it from beginning to end, being performed under real-life conditions. The meeting attended was chosen by the health professional that was participating in this study. The researcher identified the key message addressed, which defined an idea or concept to be shared, the topics that supported the development of reflection around the message, the level of message reinforcement, its specificity and depth, for which a form had to be filled out immediately after the session as a written record. Subsequently, the record was transferred to an excel sheet, for which another researcher verified the quality of the records and the communicative clarity of the records.

Afterwards, in the semi-structured interview, open-ended questions were formulated for health professionals, following the design of the episodic interview, aimed at everyday situations and events in which there is a combination of individual and collective knowledge and thinking [34]. This research project addressed the educational dimension around themes: the procedure adopted to select themes, considering the participation of different actors and the dynamics of identification, and the technical references used to develop the content of the NE groups. Interviews were audio-recorded, transcribed verbatim and translated into Portuguese.

Finally, we formulated and distributed the online questionnaire through Google Docs in such a way as to enable health professionals to provide detailed information by means of open-ended answers to questions. We requested descriptions of their social characteristics, training, length of experience on the job and work routine, as well as the characteristics of the NE groups in which they participated (Table 1).

### Analysis

We organized the information obtained from the observation forms, and transcribed interviews and online questionnaires into a database. In the systematic observation, the first author organized the topics and key messages generated by group and place, looking for regularities between the data. Subsequently, an analytical work was done to establish links between the different groups from the search for a set of similar concepts and ideas to improve the construction of key messages that reflect the level of development of the support themes during the group meeting [35]. Thus, using the thematic networks method, based on the theory of argumentation, we searched for outstanding basic themes, which when grouped would indicate more abstract ideas for later, and would be represented by global themes [36]. Once the thematic networks were organized, the second and third coauthors reviewed the clusters until a level of coherence was reached and the overall themes were rectified.

For the analyses of the information obtained during interviews, we used the technique of Collective Subject Discourse (CSD) [37, 38]. The transcriptions were analyzed by third and fourth coauthors, looking to extract the central ideas from the interviews. From different central ideas, all declarations were reorganized to construct a new synthesis discourse. This synthetic speech of CSD is written in the first person singular, which sought to express the thought of the collective as if the latter were the source of the discourse, maintaining their literality [37, 38]. The theoretical underpinning of CSD corresponds to social representations, which correspond to a set of explanations, beliefs, and ideas. The speaker of the discourse has a personal story that fits into the social context, which is ideologically marked and permeated by the diverse and heterogeneous social voices indicated that, in turn, contribute to various intersecting discursive productions [39].

Finally, to integrate all the findings and combine the methodologies, we triangulated the key messages identified and the synthetic speeches. We sought to identify the convergences of the results to improve the understanding of the phenomenon and to expand the potential of knowledge about the process of implementation of themes, although without making any judgments as to the congruence between the methods [34, 40]. The present study did not intend to seek an interpretation of cause and effect, but rather a dynamic understanding that would include access to a greater variety of explanations, beliefs, and ideas of health professionals. Thus, aiming to achieve greater reliability of findings, we used a comparative methodology, in accordance with the following procedure [27]: 1) targeting data by city; 2) generating categories of analysis by city on the basis of cities, and 3) comparing and contextualizing the findings.

## Results

We found four global themes (Table 2), two of which were common to both cities. The first common global theme was about eating habits. It should be emphasized that health professionals from São Paulo worked on more specific messages about the benefits of the intake of certain types of food, such as apples, hibiscus tea, whole grain cereals, and fibers. On the other hand, in Bogotá, the approach was aimed, in general terms, at the purchase of and properties and preparation of foods. The second global theme was about life cycle. The focus was on messages for different age groups, where childhood was an aspect identified as a priority.

**Table 2:** Nutrition education key messages organized by global themes, obtained through systematic observation of groups. São Paulo, Brazil, and Bogotá, Colombia, 2012

Global themes	Messages	
	São Paulo (*n* = 28)	Bogotá (*n* = 13)
Feeding habits	• Eat fruits, especially apples, once a day if possible • Include the consumption of whole grains in your habits • Eat fiber and drink a lot of water • Reduce the amount of oil and sugar in recipes • Come to the group to exchange recipes • Hibiscus tea can bring health benefits • Read food labels • Watch for food hygiene at home • Be aware of the size of portions in the food pyramid • Eat slowly at mealtimes • Clarify questions to improve feeding	• The street market can relate to the food groups
		• Know the properties of fruits and vegetables
		• Vary the preparations of fruits, vegetables, and legumes and share what you know • Avoid falling ill with appropriate food handling practices at home • You know more than me about food, but learn more • Identify problems and solve them with what is available to you • Get to know the food groups on the “train” to improve your eating habits
Life cycle	• Watch your weight gain during pregnancy • Weigh children • Encourage chewing through to the consistency of porridge • Avoid products with cow’s milk before their first birthday • Look for healthy food options at lunch at school	• Try new ways of practical preparations for babies, they are easy and inexpensive • Identify the characteristics of feeding in different age groups • Learn about overweight and obesity in the elderly and avoid these conditions with a healthy diet and physical activity
Disease	• Eat every 3 h and reduce the amount of food at lunch and dinner • Confront your problem • Use sucralose • Incorporate weekly tasks through nutritional re-education • The priority now is health, find time to do physical activities • Maintain the goal; there is support here	
“Being a multiplier”		• Reinforce your knowledge of food and nutrition; it is essential to advise mothers • Include what is learned in their daily lives and replicate it in the nursery community • Learn about the right to food to work with in the nursery community

The third global theme, specific of São Paulo, was about disease emergence and it was aimed at health conditions, such as diabetes, hypertension, and obesity. In this case, professionals use a more prescriptive message such as “eat every 3 hours”. In Bogotá, we found the fourth global theme. It was about “being a multiplier” and it mentioned leaders and teachers training in this territory, including instructions for the population on ways to deal with eating habits.

**Fig. 1:**
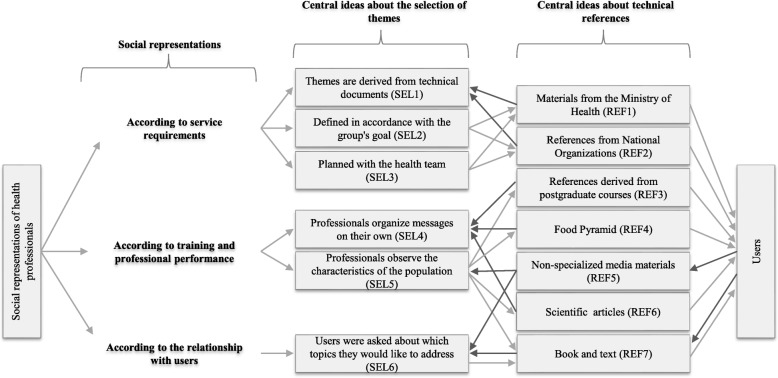
The process of implementation of themes in São Paulo, Brazil 2012**.** Note. SEL: Central ideas from the selection of themes**.** REF: Central ideas from the technical references

**Fig. 2:**
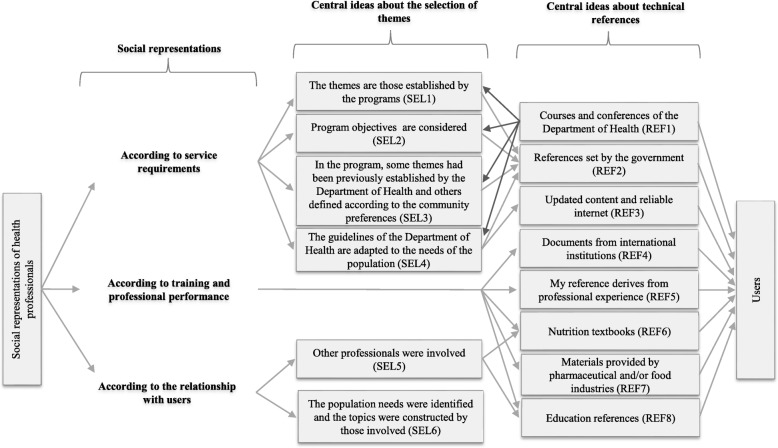
The process of implementation of the themes in Bogotá, Colombia, 2012. Note. SEL: Central ideas from the selection of themes. REF: Central ideas from the technical references

We identified six central ideas in the procedure adopted to select the themes by city and generated seven central ideas in São Paulo and eight in Bogotá relating to the technical references of messages (Figs. 1 and 2). Through the synthetic speeches constructed by each central idea, three social representations appeared, interfering with the process of implementation of themes, which dialogue with the characteristics of the messages: “the service requirements”, “the training and professional performance”, and “the relationship with users”.

### According to service requirements

For health professionals, the logic of the development of NE groups and work priorities will be controlled (Figs. 1 and 2). In Bogotá, the identification of the themes goes through different justifications and subtle variations, although there is a constant emphasis on something that has been previously decided. To illustrate this, the Fig. 2 shows the themes were established by the programs (SEL1), or were made from the objectives of the programs (SEL2). Therefore, when situations arose that required users to address issues of disease, but not framed around either promotion or prevention, it was recognized that they did not match the activities created by the NE groups and those topics were not worked on by the professionals. In the programs, the themes were specified by the DH guidelines (for example, the message on the right to food), and then presented to the community groups, always related to healthy feeding habits. Furthermore, technical resources provided to health professionals by the DH courses were perceived as important (Fig. 2).

The DH established priorities to work according to age groups, which can be recognized in the key message. However, the social representation in Bogotá stated that, regardless of the age range of users, the content stipulated should be given to them. The decisions regarding these themes were inflexible and the themes were shown as a package of services ready to be executed (SEL3, Fig. 2), which became apparent in the program, where some questions had already been established by the DH and others defined according to the population’s preferences:

*They are fixed themes that we must deal with, as they wouldn’t let us do this through an audit. They’ve been making some changes and updates on these themes since last year* (SEL3, Fig. 2).

In fact, health professionals perceived materials recommended by the government, which included documentation from the DH decrees and food guidelines (REF2, Fig. 2), as an important technical component. In São Paulo, we identified that the messages are focused on disease, thus representing a priority.

In the cases of specific diseases, such as diabetes, material published by the Brazilian Association on Diseases was used as a reference (SEL1 and REF2, Fig. 1). The question of disease also agreed with the analogous idea relating to themes dependent on the group’s goal (SEL2, Fig. 1), such as the weight loss group.

Another aspect identified was related to the promotion of physical activity, which appeared in the messages in both cities, although in São Paulo it was recommended specifically for the treatment of diseases. Thus, health professionals reported that the theme selection is not limited to one professional, but it is rather a multidisciplinary work that selects certain concepts to be approached:

*In fact, it was planned with the team, we as NASF. So, together with the physical educator, psychologist and nurse, we brought up some themes, a little about what the concept is* (SEL3, Fig. 1).

### According to training and professional performance

The relationship between health professionals and their work in NE groups reveals a pedagogical repertoire according to your professional experience and specific training (Figs. 1 and 2). In the specific case of São Paulo, the messages about the preparation for a meeting on a specific foodstuff was related to the social representation of the importance of using material aied by postgraduate courses on functional, clinical, human, or therapeutic nutrition as a reference (REF3, Fig. 1). Furthermore, the messages about food groups and portions and feeding habits, were considered, although derived from the food pyramid (REF4, Fig. 1). This pictorial representation was referred to because it helps to increase the users’ level of understanding, symbolizing the practical reality, improving adherence to specific diets and at the same time enabling people to speak the same language.

Therefore, replying to concerns expressed during a meeting satisfied the interest and desire of health professionals to listen and understand the group. Materials from non-specialized sources (REF5, Fig. 1) must be consulted to discover the origin of the information people receive and consult, whether through television, magazines, or the Internet, and the information provided by fellow workers.

Accordingly, when formulating or implementing action programs, it was critical to establish goals and to develop messages and a multimedia plan. Health professionals are given the responsibility of meeting the demands of an educational group throughout an undefined period of time. Thus, their perception of freedom in São Paulo was present in the development of the groups:

*I ended up defining it, because I think you need to start from the basics if you’re to instruct someone. So I usually chose the majority of themes* (SEL4, Fig. 1).

Concerning health professionals’ perceptions of their use of the Internet, in São Paulo, this tool was more frequently consulted to obtain better explanations and as a resource to help decision making using databases and scientific articles (REF6, Fig. 1). Conversely, in Bogotá, a social representation of distrust of information sources by health professionals was identified (REF3, Fig. 2).

In the case of Bogotá, as program executors, health professionals do not seem to have the possibility of selecting themes, because central ideas were not provided. Health professionals became program executors based on their social standing and personal references, not necessarily on their technical knowledge about nutrition. For example, the technical reference specified the importance of the professional experience; however, colleagues and health professionals themselves, regardless of their nutritional knowledge and experience, are used as a practical means to meet the needs of the group:

*Well, when I have little time, I look for colleagues, they have some experience that can increase your knowledge a little further [...] and also on the basis of one’s own experience with groups* (REF5, Fig. 2).

### According to the relationship with users

Health professionals construct interpretative variations about users (Figs. 1 and 2). In São Paulo, they recognized the importance of their suggestions, but the users are the most important:

*As we prepare a job, the group presents the need. They define the need, we make some suggestions, but in the end, they’re the ones who decide* (SEL6, Fig. 1).

Media materials are present in this context as another interface for dialogue with users because frequent questions arise due to the great focus of the media on nutrition science (REF5, Fig. 1). On the other hand, books as a source of consultation reflect the greater interest in more formal contents, which enable us to verify whether the information is scientifically correct and to delve into specific themes (REF7, Fig. 1 and REF6, Fig. 2).

Furthermore, the technical references extracted from the literature showed that health professionals mentioned children as an important topic. The variety of books and topics on clinical pediatrics are in agreement in both cities. Paradoxically, the industry of breast milk substitutes affected health professionals’ references, as opposed to the strategy of groups that promote breastfeeding, working in Bogotá:

*Supposing what you have is some material about breastfeeding provided by a Lab; they give you some specific tips that I work on with the population* (REF7, Fig. 2).

In Bogotá, beyond user participation, other actors from other institutions were perceived as important. The work is an interaction among institutions through the intersection of the approach of a certain type of user. The social representations indicated that the decisions regarding themes appeared together with a trend toward the vertical relationship of health professionals:

*According to the pre-test, we say which disability exists, we speak to the teachers, they help us choose the topics or the topics are chosen by doctors, who decide on theme plans* (SEL5, Fig. 2).

Among Bogotá’s health professionals, public participation in the choice of themes takes place through the identification of the population needs, which is a technique facilitated by the intervention of a social assistant. There are two particular messages from Bogotá: the need to appreciate the knowledge of others, because the majority of users in the NE groups were older, and the recognition of the population needs even before starting the programs, this being related to the technique of identifying needs as brokered by a social assistant, mentioned above. Thus, procedures for the identification of needs can also be represented as a tool to facilitate negotiation between health professionals and the population (SEL6, Fig. 2).

Finally, there was a direct connection between how to work with users, teachers and leaders and what was submitted by health professionals in their social representations of technical references, which was the perception of how to overcome the difficulties of working with the population:

*Working in the field is very difficult, but I believe that what we are doing is a bit like Freire in his book “Popular Education for Community Work”* (REF8, Fig. 2).

## Discussion

This article presents the results of a qualitative comparative study, with the aim of gaining a better understanding of the process of implementation of the themes discussed by health professionals in the NE groups in two PHC models. Although there is no intention to generalize these findings to other NE groups that exist in São Paulo and Bogotá, the social representations highlight the health service, the training and performance professional, and the relationship with users in this process.

Two notions have been identified that shape the process of implementation of themes. First, there is the notion of control. The service requirements probably centralized the themes because of the configuration of the PHC service with the health system. In São Paulo, the themes discussed focus on the great demand for treating chronic diseases. Although what is expected from the PHC is health prevention and promotion, rather than the old model that prioritized cure [41], this does not seem to make sense for the interaction between PHC and the Brazilian Unified Health System (SUS) proposal. PHC services must meet the demand for universal coverage, and they are the first contact with the system so that the logic follows an approach that is more resolutive than preventive and does not seek to separate health promotion and disease prevention from health care. Additionally, the emphasis on disease in nutrition in PHC has been debated. In England, limited experiences have been mentioned in the intentional approach of themes on nutrition in PHC services, because these are not organized, but instead tend to pass unnoticed. Moreover, because the number of nutritionists is small, the interest is restricted to the treatment of the disease and the creation of a treatment plan [42].

Conversely, in Bogotá the focus of themes is on health promotion. In fact, in Colombia, PHC is not supported by its own public policy and, as a result, it is fragmented and disconnected from the health system. Being a particular case, Bogotá provides PHC services from the public sphere. It combines promoting, preventive, curative and rehabilitative actions in its comprehensive approach [32]. Thus, themes related to disease treatment in the logic of the groups studied corresponded to other service levels or they are explicit objectives of the private market.

Secondly, there is the notion of specificity. The training and performance professional and the relationship with health system users remind us of the complications associated with the process of implementation of themes. In São Paulo, the creation of groups by health professionals stands out, with a longitudinal follow-up and undefined duration. Part of the social representations of health professionals relates to their joint responsibility with the users to establish themes that possibly express the appropriation of the participatory pedagogical model by actively involving users [12, 24]. The adoption of this model is highly influenced by the guidelines of the national policy for health promotion [30].

In contrast, the profile of the short course groups in Bogotá takes into consideration the fact that health professionals have a connection with users from groups of popular initiative, even though their interaction is restricted. The function of the groups was designed to meet a higher priority for information rather than education, partly following the methodological proposal that recognizes the importance of communication and social support [43]. The social representations corresponded to a pre-established procedure of selection of themes, close to a prescriptive model, with the decision made by the centralized DH health professionals. In this city, the conceptual focus is based on the socio-ecological model that includes positive health, critical theory and constructivism, although the theoretical position of health education in national policies related to NE is not clearly identified [30]. Interventions in megacities necessarily have to adopt a more flexible approach that enables one to deal with the coexistence of extreme conditions [44]. In fact, the use of manuals or protocols in the services can strengthen health professionals, while at the same time rationalizing care to the most, causing inflexibility of care because it standardizes it and, consequently, does not enable the inclusion of users’ specific health needs [45].

Nevertheless, some contradictions were revealed in the present study when health professionals of both cities said, “You have to start from the basics” and “We say which disability exists”. These sentences express a social representation of the population’s supposed ignorance, which contrast with the presumption of the popular education proposed by policies in Brazil or the constructivism mentioned in Bogotá [46].

Another incoherence found was the understanding of autonomy. In São Paulo, health professionals’ freedom sometimes leaves room for some distortion in planning. And in Bogotá, their autonomy is understood to express itself in their training of the population for citizenship and empowerment, but “strangely” this does not apply to the health professionals themselves, perhaps because of job insecurity. Unfortunately, in Bogotá, there is a high turnover of outsourced labor, where short fixed-term contracts are used, leading to a lack of continuity of the program vis-à-vis the population [47].

In addition, specificity can be intermediated by the level of development of discussions in the area, in the process about technical references. There is a greater market for postgraduate courses in São Paulo, which go against the media discourses, because they interact with services that require disease approaches. Furthermore, in the case of the health professionals interviewed, most of them had a postgraduate degree in a clinical area, rather than PHC. In Brazil, studies show that health professionals did not unanimously endorse the government’s recommendations, when the media was less favorable [48]. Another source of information is the Internet, which can be valuable for updating information; it is, nevertheless, necessary to critically analyze information from the Internet and know how to choose one’s sources [49].

In contrast, in Bogotá, the availability of postgraduate courses is limited, so that the service is the provider of technical references for the context of PHC. Professional experience is also reported as constituting another source, in the absence of sufficient theoretical and scientific knowledge, where the majority of health professionals referred to their professional experience, when addressing food issues, and to their dependence on educational strategy in their discussions with users [50].

We had some limitations during the data collection in Bogotá, because a change in the city’s administration led to the temporary interruption of some groups participating in NE. Thus, it was only possible to take one part of the program into consideration, the one corresponding to the community; the professional and educational approaches promote healthy eating, but are not observed. Despite these limitations, we identified some trends in both cities that converge with the tensions found in terms of service quality. On the one hand, there is a massive production that encourages the quantification of work of health professionals, whereas on the other hand, services require health professionals who can provide personalized services, within a holistic perspective. In this conflict, many health professionals lose their motivation and value [51].

## Conclusions

The findings presented here suggest that the process of implementing the themes discussed by health professionals in NE groups reveal two mechanisms to approach eating, including the appropriations of communication and educational models that appear in the services. Depending on these appropriations, the development of educational messages with users can be more decentralized and organized, which is in agreement with service control and its relationship with administrative models that are increasingly closer to outsourcing and privatization of that which is public. Additionally, this could be a more mobilizing task, which falls into the sphere of specificity, a result of the bond between professional and user. Thus, health education, which seems to be homogeneous for the qualification of health professionals, unfolds into several roles played by health professionals in the workplace with the themes on healthy eating, as part of NE. Our aim is to add more coherence between the educational model planned and the effectiveness of actions developed by health professionals in the context of PHC. This study contributes to the discussion about educational models in health care and their effects on the qualification of health professionals within public health services, especially on those who work in low- and middle-income environments. Other studies should be undertaken to better understand NE, especially with regard to the assessment of users’ perceptions.

## Abbreviations

BTA: Bogotá
CSD: Collective Subject Discourse
DH: Department of Health
LAMICs: Low- and Middle-Income Countries
NASF: Family Health Support Center
NE: Nutrition Education
PHC: Primary Health Care
PIC: Collective Intervention Plan
PSF: Family Health Program
REF: Central ideas from the technical references
SEL: Central ideas from the selection of themes
SP: São Paulo
SUS: Unified Health System
